# Perceptions of “Healthy Life Expectancy” of Individuals With Diseases: An Online Survey in Japan

**DOI:** 10.1002/hsr2.71533

**Published:** 2025-11-26

**Authors:** Kyunghee Lee, Kazumi Ota, Tetsuya Toma, Masako Toriya

**Affiliations:** ^1^ Keio University Global Research Institute Keio University Tokyo Japan; ^2^ Graduate School of System Design and Management Keio University Yokohama Japan; ^3^ Department of Innovation Science School of Environment and Society, Institute of Science Tokyo Tokyo Japan

**Keywords:** colorectal cancer, dialysis, healthy life expectancy, kidney failure, online survey, satisfaction with life scale (SWLS)

## Abstract

**Background and Aims:**

In Japan, discussions around healthy life expectancy (HLE) are often focused on prevention of diseases or disabilities. However, the advancements in medical technologies have led to an increase in the number of individuals living with these conditions. The aims of this study are (1) to compare the perceptions of HLE between individuals with and without a disease, and (2) to explore the factors associated with attaining HLE among those who have diseases.

**Methods:**

An online questionnaire was administered to Japanese individuals which included patients with colorectal cancer (cancer group), individuals undergoing dialysis for kidney failure (dialysis group), and those without diseases (ND group). ANOVA multiple comparisons and the Tukey‐Kramer post hoc test were conducted among the three groups to examine whether there are differences in the perception of HLE between individuals with and without diseases. Binary logistic regression analysis was performed to identify factors related to achieving HLE among individuals with diseases.

**Results:**

A total of 626 participants responded, with 208 in the cancer group, 210 in the dialysis group, and 208 in the ND group. The dialysis group was more likely to perceive that their health problems affect their daily lives and less likely to attribute the attainment of HLE to individual effort or family effort compared to the cancer and ND groups. Among individuals with diseases (cancer and dialysis groups), being female, having cohabitants, and having a higher SWLS score were significantly associate with achieving a HLE.

**Conclusion:**

Life satisfaction is significantly associated with the belief in achieving HLE despite physical or mental challenges among those living with a disease. Ameliorating life satisfaction through social participation and social support as well as psychological interventions can be one of the contributing factors for extending HLE.

## Summary

- This study aims to investigate by an online survey the perceptions of healthy life expectancies among Japanese individuals with colorectal cancer, those undergoing dialysis for kidney failure, and those without diseases.
- Multiple regression analysis revealed a significant association between a higher Satisfaction with Life Scale score and the perception that a healthy life expectancy could be achieved despite health challenges.
- Ameliorating life satisfaction through social participation and social support as well as psychological interventions can be one of the contributing factors for extending healthy life expectancy.

## 1 Background

Increased life expectancy has been observed worldwide; between 2000 and 2019, the global life expectancy has increased from 66.8 years to 73.1 years (9.4%) [1]. However, increased longevity does not always equate to longer healthy life. Among 183 World Health Organization (WHO) member states, the gap between lifespan and healthspan (i.e., years lived in good health) widened from approximately 8.5 years in 2000 to about 9.6 years in 2019 [2]. The largest healthspan‐lifespan gaps in 2019 were observed in the United States (12.4 years), Australia (12.1 years), and New Zealand (11.8 years) [2]. When evaluating quality of life, the emphasis is shifting more toward achieving healthy longevity because the healthspan‐lifespan gap can reflect the extent of lifespan burdened by disease [3]. In Japan as well, extension of healthy life expectancy (HLE) has been emphasized. Healthy Japan 21 (2000) and its second phase (2012) identified the extension of HLE as a significant goal [4, 5], and narrowing the gap with overall life expectancy is recognized as vital for maintaining the population's quality of life and ensuring the sustainability of the social security system [5].

HLE is “the average number of years that a person can expect to live at a certain level of health” [5] p.150. There are several types of HLE including disability‐free life expectancy, disease‐free life expectancy, quality‐adjusted life expectancy, self‐rated healthy life expectancy, disability‐adjusted life expectancy, and health‐adjusted life expectancy (HALE) [6]. WHO uses HALE, which is calculated by assigning weights to various kinds and severity of unhealthy status for each individual, while Japan and most Western countries use disability‐free life expectancy, which reflects the presence or absence of a disability [5, 6]. According to WHO, in 2019 Japan exhibits the longest life expectancy in the world (84.5 years) and the second longest HALE (73.4 years) after Singapore [7]; however, their gap has also been widening like many other countries in the world; 10.4 years in 2000 and 10.9 years in 2019 [7]. On the other hand, the data from Japanese Mistry of Health, Labor and Welfare [8] indicate that the average gap between life expectancy and HLE among Japanese population were 10.48 years in 2001 and 10.40 years in 2019, showing slight decrease in two decades. The discrepancy between the data from WHO and MHLW is probably due to the different concept of HLE used in each organization; WHO uses HALE while Japanese MHLW adopts disability‐free life expectancy [5].

In Japan, HLE is calculated based on the Comprehensive Survey of Living Conditions, a large‐scale survey targeted at Japanese citizens conducted every 3 years [9]. The primary indicator considers the response to the question, “Are you currently affected in your daily life owing to health problems?” Those answering “No” and “Yes” are classified as healthy and unhealthy, respectively. As a secondary indicator, “good,” “fairly good,” and “normal” responses to the question, “How is your current health condition?” are considered healthy, while “not so good” and “not good” responses are deemed unhealthy.

HLE is often described as “the period during which medical care and long‐term care are not required,” creating the perception that individuals with diseases or disabilities cannot attain HLE. Initiatives to “extend healthy life expectancy by 3 years or more,” as outlined in the “Summary of the Headquarters for Social Security and Workplace Reform Looking Ahead to 2040” [10], include measures for “disease prevention and prevention of serious disease,” “long‐term care prevention and frailty prevention,” and “dementia prevention.” However, advancements in medical technologies have led to an increase in the number of individuals with diseases. For instance, the Foundation for Promotion of Cancer Research reports a rise in the number of cancer patients in Japan since 1985, maintaining a steady level in recent years. Simultaneously, the cancer mortality rate has consistently declined from 2005 to 2020 [11]. This global trend is reflected in the average annual age‐standardized incidence rates for all cancers combined, which increased in 123 of 195 countries from 2007 to 2017. Conversely, the average annual age‐standardized mortality rates for all cancers combined declined in 145 of 195 countries [12]. In 2017, approximately 700 million individuals worldwide had chronic kidney disease at all stages [13], surpassing the numbers with diabetes and chronic obstructive pulmonary disease [13, 14], and the population requiring dialysis is on the rise [15]. Furthermore, a meta‐analysis of 126 studies of approximately 15.4 million adult populations in 54 countries found a global multi‐disease prevalence rate of 37.2% [16]. To extend HLE for everyone, it is necessary to focus not only on the prevention of diseases and disabilities but also on the ever‐increasing number of individuals living with these conditions.

Previous studies on HLE in Japan were mainly observational studies on the factors related to HLE such as biological factors, lifestyle habits, and psychological, social, and environmental factors contributing to the extension of HLE [17, 18, 19, 20, 21, 22, 23, 24]; intervention studies at community and societal level aimed at maintaining and preventing deterioration of physical and mental health [25, 26]; methodological approach to calculate HLE [27, 28, 29]; and analysis on the past and current trends of life expectancy and HLE [30, 31]. However, there are limited number of studies exploring perception of HLE of individuals living with diseases or disabilities in Japan. The present study has two objectives: (1) to compare the perceptions of HLE between individuals with and without a disease, and (2) to explore the factors associated with attaining HLE among those who have diseases.

## 2 Methods

### 2.1 Study Population and Study Design

In October 2023, an online survey was conducted through an online research company (MACROMILL, INC.), which recruits a wide range of registered panelists, including those with various diseases. Panelists were free to participate in various surveys conducted by the company and received points that can be converted into cash or other rewards from the company. The amont fo rewards received by the panelists from the research company are not affected by how they respond. Therefore, it is believed that response biases in this online survery are minimal. In Japan, several large online surveys have been reported and planned by companies to investigate the awareness of individuals with diseases [32, 33, 34]. Although it is ideal to include patients with all kinds of diseases and disabilities in the study, due to the cost and feasibility of the online survey, we only selected patients with colorectal cancer and those with kidney failure undergoing dialysis to represent people with diseases and disabilities. We selected these patients because cancer and kidney failure are two of the 10 most common causes of death in Japan in 2023 [8]. Among cancers, colorectal cancer had the highest number of cases for men and women combined in Japan in 2023 [35]. Patients requiring dialysis bear a significant burden in terms of time and effort spent on treatment, which is believed to have a major impact on their daily lives [36, 37, 38]. For these reasons, this study selected colorectal cancer patients and dialysis patients as the disease groups and chose a healthy group (ND group) as the comparison group. To ensure a sufficient number of responses and facilitate the interpretation of the results, the selection criterion for the cancer group was having received treatment for colorectal cancer (surgery, drug therapy (chemotherapy), or radiation therapy) within the past 3 years. The selection criterion for the dialysis group was current dialysis treatment. The selection criterion for the ND group was not to have a current diagnosis of a disease or disability. The exclusion criteria for all three groups were as follows: age < 20 years, diagnosis of dementia or psychiatric disorders, and diagnosis of colorectal cancer and dialysis. Since the age range of respondents in the cancer and dialysis groups was expected to be higher than that of the ND group, we divided each group into eight categories: four categories for generation (40 s, 50 s, 60 s, and 70 s or older) and two for sex to keep the age and sex of all groups at the same level. The respondents were divided into eight categories, 25 in each category and 200 in each group (600 respondents in total), which were the targets of this study. Through an online research company, those registered as colorectal cancer patients, those registered as chronic kidney patients, and those registered as having no disease were invited to respond to the questionnaire via e‐mail until the number of respondents reached the number of those assigned to this study. If equal allocation to each group could not be achieved, adjustments were made by increasing the number of other cells to ensure the expected total number of respondents in each group. To exclude as many poor or dishonest responses as possible, the top 3% of responses with the shortest response times were excluded according to the regulations of the online survey company. Furthermore, after receiving data from the online survey company, the authors visually checked the responses of all the respondents to ensure that there were no unnatural responses, such as answering the same number of questions.

A pre‐survey screening survey was administered to participants, preceded by clear communication of the survey's nature and purpose. Participants were notified about the inclusion of certain personal information, the exclusive use of responses for study analysis, the survey's affiliation with a specific company, the anonymity of responses, and the significance of providing informed consent. Both screening and primary surveys mandated complete responses, allowing participants to opt out at their discretion. Valid responses addressing all items. Ethical approval for this study was granted by the Keio University Research Ethics Review Committee in September 2023 (23‐013).

### 2.2 Questionnaire

The survey questionnaire comprised 31 items (32 items, including the cancer stage item for the cancer group) from Q1 to Q6 (Supplementary Table 1).

- Q1, Demographics (four items) (five items including cancer stage items for the cancer group only): Items related to respondents' attributes, such as educational attainment and cohabitants (s), were examined.
- Q2, Attainment of HLE (one item): In this study, attaining HLE is defined as answering “no” to this question. This question is same as the question used in a survey administered by the Japanese government to determine HLE of Japanese population [9]. This item queries, “Are you currently affected in your daily life because of health problems?” and the choices are “Yes” or “No.”
- Q3, SOC‐13 (13 items): The Sense of Coherence (SOC), proposed by Aaron Antonovsky [39], is a central concept in his Salutogenic Model, which focuses on factors that promote health and well‐being rather than just preventing diseases [40]. SOC explains how individuals perceive and cope with stress and challenges in life, determining their ability to maintain or improve health. A strong SOC enables individuals to view stressors as manageable and meaningful, helping them maintain resilience and move toward better health, even in difficult circumstances [40, 41].
  A questionnaire to measure SOC was developed and validated by Antonovsky [42, 43]. Although the original version consisted of 29 questions (SOC‐29), the shorter version of 13 questions (SOC‐13) was also developed by Antonovsky [39], and the reliability and validity of the Japanese version of the SOC‐13 were verified by Togari et al. Togari et al., [44]. The score of SOC‐13 ranges between 13 and 91 points (each question being scored on a 7‐point Likert scale), with higher scores indicating a stronger SOC. According to the prior study conducted on 4,000 Japanese men and women aged 25 to 74, the average scores on SOC‐13 was 59.0 [45]. Although no differences in scores were observed based on gender or place of residence, the SOC scores tended to increase with age (the average score for those aged 65 to 74 was 63.9) [45].
  The previous studies using SOC with clinical samples reported a negative association between SOC scores and distress in cancer patients [46], frequencies and levels of stressful events in women with breast cancer [47], and denial, behavioural disengagement, venting and self ‐blame in persons with chronic heart disease [48]. A positive association was also reported with self‐rated health status and QOL in women with breast cancer [47] and acceptance in persons with chronic heart disease [48]. The SOC was used in this study because we hypothesized from these prior studies that SOC scores can be associated with the attainment and perception of HLE in the cancer and dialysis groups.
- Q4. Satisfaction with Life Scale (SWLS) (5 items): This scale specifically evaluates an individual's global cognitive judgments of their life satisfaction, rather than focusing on specific domains (e.g., health or work) [49] and is one of the most widely used measures of life satisfaction. The Japanese version was developed by Sumino [50]. The SWLS employs a 7‐point Likert scale (1 = strongly disagree to 7 = strongly agree) with scores ranging from 5 to 35, with a score of 20 representing the neutral point on the scale. Scores between 5 and 9 indicate that the respondent is extremely dissatisfied with life, scores between 15 and 19 indicate slightly dissatisfied, 21 and 25 indicate slightly satisfied, and scores between 31 and 35 indicate extremely satisfied [51].
  The SWLS has been used with clinical samples such as cancer [52], Parkinson's disease [53], spinal cord injuries [54, 55] and psychiatric patients [56] as well as nonclinical community dwelling populations worldwide (e.g. [57, 58, 59]). We chose to use this measure because prior studies have used it to examine the relationship between elders' subjective well‐being and physical health (Minagawa & Sato, 2022), self‐rated health [60], and perceived level of stress [61]. We hypothesized that scores on SWLS are associated with the attainment and perception of HLE in the cancer and dialysis groups.
- Q5: Awareness of healthy life expectancy (1 item): To explore the level of awareness of the phrase “healthy life expectancy,” we asked the following four questions: Familiar with its meaning, Somewhat familiar with its meaning, Have heard of it but not familiar with its meaning, Never heard of it.
- Q6, Perception of HLE (7 items): To explore how individuals perceive HLE, we made original questions focused on the rainbow model of social determinants of health [62] We asked how much they thought the following items were needed to achieve HLE as defined by the MHLW: individual efforts (Q6‐1), family efforts (Q6‐2), efforts of friends (Q6‐3), efforts of companies/educational institutions (Q6‐4), and efforts of national/local governments (Q6‐5). In addition, we asked whether they thought HLE could be achieved despite physical (Q6‐6) or mental (Q6‐7) diseases or disabilities. Choices were made on a 7‐point scale.

### 2.3 Variable Processing

The one respondent who answered “other” in the item related to the educational level (Q1‐2) was considered equivalent to “technical college/junior college” because “junior college” was written in the free answer space. Reverse item processing was performed to facilitate the interpretation of the results for questions Q2–1, Q4–1 to 4–5, Q5–1, and Q6–1 to 6–7. For attainment of HLE (Q2–1), individuals who responded “Yes” were defined as the non‐HLE sub‐group, and those who responded “No” were defined as the HLE sub‐group. For the SOC‐13 (Q3–13) and SWLS (Q4–1 to 4–5), each item was scored respectively based on each previous study [39, 49]. For the perception of HLE (Q6–1 to 6–7), four points were considered as the median score for the choices.

### 2.4 Statistical Analysis

All statistical analyses were conducted using Bell Curve for Excel version 4.05 (Social Survey Research Information Co. Ltd, Tokyo, Japan). To verify reliability, Cronbach's α coefficient was calculated for 27 perception‐related question items (Q2 to Q6). The chi‐square test was used to compare the proportions of gender, age groups, place of residence, educational background, presence of someone to consult with, and attainment of HLE among the three groups (cancer, dialysis, and ND) (*p* < 0.05). Analysis of variance (ANOVA) was used to compare SOC‐13 scores and SWLS scores among the three groups (cancer, dialysis, and ND) (*p* < 0.05).

To examine whether there are differences in the perception of HLE between individuals with and without diseases, ANOVA multiple comparisons and the Tukey‐Kramer post hoc test were conducted to compare HLE perceptions (Q5–1, Q6–1 to Q6–7) among the three groups (cancer, dialysis, and ND) (*p* < 0.05). Based on the results obtained from the between‐group comparison, an exploratory analysis was conducted by creating histograms for the responses to Q6–6 and Q6–7.

To identify factors related to achieving HLE among individuals with diseases, binary logistic regression analysis was performed, targeting the disease groups (cancer and dialysis). In this analysis, attainment of HLE was set as the dependent variable, while gender, age, place of residence, educational background, presence of someone to consult with, caregiving status, SOC score, and SWLS score were used as explanatory variables (*p* < 0.05). Based on the results of the logistic regression analysis, exploratory analysis was conducted by visualizing SWLS scores for both the non‐HLE group and the HLE group within each of the three groups (cancer, dialysis, and ND) using histograms.

## 3 Results

### 3.1 Response Tendency and Reliability of the Questionnaire

A total of 626 respondents participated in the study, divided into three groups: cancer group (208 participants), dialysis group (210 participants), and ND (non‐disease) group (208 participants). Demographic details are presented in Table 1. Significant differences in the proportion of participants among the three groups were observed for gender and place of residence. Additionally, males in their 50 s accounted for 26.6% of the dialysis group sample (Supplementary Table 2). Regarding attainment of HLE, the number of participants who reported experiencing difficulties in daily life was highest in the dialysis group (152 participants, 72.4%), followed by the cancer group (88 participants, 42.3%), and lowest in the ND group (27 participants, 13.0%). Significant differences were observed in the proportion of participants among the three groups (*p* < 0.001). The SWLS score was lowest in the dialysis group (16.16 ± 7.46) compared to the cancer group (18.75 ± 6.92) and the ND group (19.26 ± 6.90), with significant differences among the three groups (*p* < 0.001). The overall SOC score of the respondents was an average of 53.50 points (SD ± 6.69), with no significant differences observed among the three groups. The Cronbach's α coefficient for the 27 items was 0.75.

**Table 1:** Demographics of the subjects.

		Total *n* (%)	Cancer group *n* (%)	Dialysis group *n* (%)	ND group *n* (%)	*p*
Sex^a^	Male	371(59.3)	107(51.4)	160(76.2)	104(50.0)	*p* < 0.001**
	Female	255(40.7)	101(48.6)	50(23.8)	104(50.0)	―
Age group^a^	40 s	136(21.7)	43(20.7)	41(19.5)	52(25.0)	0.4
	50 s	179(28.6)	57(27.4)	70(33.3)	52(25.0)	―
	60 s	166(26.5)	56(26.9)	58(27.6)	52(25.0)	―
	70 s+	145(23.2)	52(25.0)	41(19.5)	52(25.0)	―
Cancer stage	Stage 0	―	44(21.2)	―	―	―
	Stage 1	―	45(21.6)	―	―	―
	Stage 2	―	48(23.1)	―	―	―
	Stage 3	―	34(16.3)	―	―	―
	Stage 4	―	24(11.5)	―	―	―
	Unknown	―	13(6.3)	―	―	―
Living^a^	With someone	476(76.0)	165(79.3)	143(68.1)	168(80.8)	0.0040**
	Alone	150(24.0)	43(20.7)	67(31.9)	40(19.2)	―
Educational attainment^a^	Junior high school	20(3.2)	5(2.4)	8(3.8)	7(3.4)	0.4
	High school	199(31.8)	63(30.3)	75(35.7)	61(29.3)	―
	Vocational school	65(10.4)	17(8.2)	29(13.8)	19(9.1)	―
	Technical college/junior college	82(13.1)	32(15.4)	22(10.5)	28(13.5)	―
	University	241(38.5)	85(40.9)	69(32.9)	87(41.8)	―
	Graduate school	19(3.0)	6(2.9)	7(3.3)	6(2.9)	―
	Other	0(0)	0(0)	0(0)	0(0)	―
Has someone close to talk to^a^	Yes	508(81.2)	173(83.2)	164(78.1)	171(82.2)	0.4
	No	118(18.8)	35(16.8)	46(21.9)	37(17.8)	―
Currently taking care of^c^	Child	84(13.4)	30(14.4)	14(6.7)	40(19.2)	―
	Paretnt/grandparent	60(9.6)	28(13.5)	15(7.1)	17(8.2)	―
	Spouse	8(1.3)	1(0.5)	5(2.4)	2(1.0)	―
	Other	8(1.3)	3(1.4)	1(0.5)	4(1.9)	―
	Nobody	480(76.7)	154(74.0)	178(84.8)	148(71.2)	―
Attainment of HLE^a^	No attainment of HLE	267(42.7)	88(42.3)	152(72.4)	27(13.0)	*p* < 0.001**
	(Non‐HLE sub‐group)					
SOC‐13 scores^b^	Mean, SD	53.50 ± 6.69	52.73 ± 6.92	53.63 ± 6.54	54.15 ± 6.57	0.091
SWLS scores^b^	Mean, SD	18.05 ± 7.22	18.75 ± 6.92	16.16 ± 7.46	19.26 ± 6.90	*p* < 0.001**

^a^ Chi‐squared test.

^b^ ANOVA.

^c^ Since multiple selections are possible, no statistical tests are performed

*p < 0.05

** *p* < 0.01.

### 3.2 Awareness of HLE and Perception of HLE

The mean score of awareness of HLE was greater than 3 points for the three groups, and the analysis of variance and multiple comparisons showed no significant differences among the three groups (*p* = 0.45 0.88). The results of analysis of variance and multiple comparisons among three groups on whether HLE is achieved through the efforts of “individual (Q6–1),” “family (Q6–2),” “friends (Q6–3),” “companies/educational institutions (Q6–4),” and “national/local governments (Q6–5)” showed significant differences for “individual (Q6–1)” and “family (Q6–2).” The mean of “individual (Q6–1)” was 4.83 for the cancer group, 4.32 for the dialysis group, and 5.00 for the ND group, which was significantly lower for the dialysis group than for the other two groups (*p* < 0.001). The mean of “family (Q6–2)” was 4.47 for the cancer group, 4.12 for the dialysis group, and 4.55 for the ND group, with the dialysis patients significantly lower than the other two groups (*p* = 0.02, *p* = 0.0025) (Table 2). No significant differences were found among the three groups regarding whether they thought a HLE could be achieved despite physical or mental disease or disability (Q6–6, 7) (Table 2). A histogram was created to visualize the responses of the three groups regarding whether they believe it is possible to achieve HLE despite having a disease or disability (Q6–6, Q6–7). The results showed that, compared to the ND group, a certain number of participants in the disease groups (cancer and dialysis) responded “Do not think so at all,” suggesting a potential difference in perception. This tendency was particularly noticeable in the dialysis group, which exhibited this pattern for both questions (Supplementary Figure 1) (Table 3).

**Table 2:** Comparison of awareness and perception of HLE among groups.

	Level	Group	*n*	Mean	SD	Levels	*p* value
Q5‐1 Awareness of healthy life expectancy	1	Cancer	208	3.13	0.84	1–2	0.5
	2	Dialysis	210	3.03	0.81	1–3	0.8
	3	ND	208	3.07	0.80	2–3	0.9
Q6‐1 Healthy life expectancy can be achieved by individual efforts	1	Cancer	208	4.83	1.28	1–2	*p* < 0.001**
	2	Dialysis	210	4.32	1.55	1–3	0.4
	3	ND	208	5.00	1.31	2–3	*p* < 0.001**
Q6‐2 Healthy life expectancy can be achieved by family efforts	1	Cancer	208	4.47	1.32	1–2	0.02*
	2	Dialysis	210	4.12	1.33	1–3	0.8
	3	ND	208	4.55	1.24	2–3	0.0025**
Q6‐3 Healthy life expectancy can be achieved by efforts of friends	1	Cancer	208	3.51	1.49	1–2	0.9
	2	Dialysis	210	3.46	1.50	1–3	*p* > 0.99
	3	ND	208	3.51	1.43	2–3	0.9
Q6‐4 Healthy life expectancy can be achieved by efforts of companies/educational institutions	1	Cancer	208	3.75	1.43	1–2	0.4
	2	Dialysis	210	3.58	1.38	1–3	0.9
	3	ND	208	3.79	1.39	2–3	0.3
Q6‐5 Healthy life expectancy can be achieved by efforts of national/local governments	1	Cancer	208	3.95	1.41	1–2	0.8
	2	Dialysis	210	3.86	1.55	1–3	1.0
	3	ND	208	3.91	1.43	2–3	0.9
Q6‐6 Healthy life expectancy can be achieved despite having a physical disease or disability	1	Cancer	208	4.13	1.46	1–2	0.6
	2	Dialysis	210	3.99	1.60	1–3	0.9
	3	ND	208	4.07	1.52	2–3	0.8
Q6‐7 Healthy life expectancy can be achieved despite having a mental disease or disability	1	Cancer	208	3.95	1.38	1–2	0.8
	2	Dialysis	210	3.87	1.57	1–3	0.8
	3	ND	208	3.86	1.48	2–3	*p* > 0.99

*Note:* ANOVA multiple comparison and Tukey‐Kramer post‐hoc test were performed.

* *p* < 0.05

** *p* < 0.01.

**Table 3:** Factors associated with attainment of HLE among disease groups (cancer and dialysis group).

	Univariate analysis				Multivariate logistic regression			
	Odds ratio	95% CI		*p*	Odds ratio	95% CI		*p*
Gender (Female)	1.64	1.10	2.45	0.02*	1.80	1.17	2.77	0.0071**
Age (years old)	1.01	0.99	1.03	0.18	1.00	0.98	1.02	0.83
Residence (Coresident)	1.96	1.24	3.12	0.004**	2.39	1.43	4.00	*p* < 0.001**
Educational attainment	1.01	0.88	1.16	0.88	1.01	0.87	1.17	0.89
Has someone close to talk to (yes)	0.91	0.56	1.48	0.71	0.50	0.28	0.88	0.02*
Caregiving (not required)	1.32	0.81	2.16	0.26	1.66	0.96	2.86	0.07
SOC‐13 scores	1.02	0.99	1.05	0.13	1.00	0.97	1.04	0.90
SWLS scores	1.06	1.03	1.09	*p* < 0.001**	1.07	1.03	1.10	*p* < 0.001**

*Note:* Nagelkerke R²: 0.12. The percentage of correct classifications: 64.35%.

* *p* < 0.05

** *p* < 0.01.

The results of the binary logistic regression analysis, with attainment of HLE as the dependent variable in the disease groups (cancer and dialysis), showed that being female (OR = 1.8, 95% CI: 1.17–2.77), having cohabitants (OR = 2.39, 95% CI: 1.43–4.00), and higher SWLS score (OR = 1.07, 95% CI: 1.03–1.10) were significantly associated with responding “No difficulty in daily life due to health problems” (*p* < 0.01). On the other hand, having someone close to talk to was significantly associated with responding “Difficulty in daily life due to health problems” (OR = 0.5, 95% CI: 0.28–0.88, *p* = 0.02). The correlation coefficients among the 8 variables used in the binary logistic regression analysis ranged from −0.16 to +0.28.

The distribution of SWLS scores for the non‐HLE and HLE subgroups in the three groups is depicted in a histogram (Supplementary Figure 2). 26.1% of the non‐HLE subgroup in the cancer group and 25.0% of the dialysis group had higher SWLS scores than the average SWLS score among general Japanese population (21.99) reported in a previous study [50].

## 4 Discussion

This study aimed to examine the perceptions of individuals living with diseases or disabilities regarding HLE, and to explore the factors associated with the attaining HLE (i.e., answering “no” to the question “Are you currently affected in your daily life because of health problems?”) among those who already have diseases. To the best of our knowledge, this is the first study to explore perception of HLE of individuals living with diseases in Japan. A key findings are as follow. (1) Dialysis group is more likely than cancer and ND groups to perceive that their health problems affect their daily lives. Regarding the perception of “Whose efforts do you think contribute to achieving a HLE?” the dialysis group was less likely to attribute it to individual effort or family effort compared to the cancer and ND groups. (2) Among individuals with diseases (cancer and dialysis groups), being female, having cohabitants, and having a higher SWLS score are significantly associate with achieving a HLE, and (3) Furthermore, within the disease groups, those who attain HLE have higher SWLS socores than the average score of Japanese general population [50].

This study's results indicate that 42.7% (267 respondents) of the participants reported daily life disruptions due to health problems, exceeding 17.43% among Japanese individuals over 40 years, based on the 2022 National Living Basic Survey by MHLW [9]. This discrepancy is likely due to the balanced representation of respondents from both the diseased and healthy groups in our study. Comparing only the ND group (13.0%), the results aligned with those of the 2022 National Living Basic Survey by the MHLW. Therefore, this study obtained responses from a generally valid group. The reliability of the 27 items is sufficient with a Cronbach's alpha of 0.75 [63, 64].

The present study indicates that the dialysis group is more likely than the cancer and ND groups to perceive that their health problems affect their daily lives, and less likely to believe that HLE could be achieved through individual effort or family effort. Regarding the perception of whether HLE can be achieved despite having a disease or disability, some individuals in the disease group expressed a strongly negative view toward achieving HLE, with this tendency being more pronounced among dialysis patients. Patients undergoing hemodialysis typically require treatment that involves visiting dialysis centers two to three times a week, with each session lasting three to 4 h [38]. These factors place a substantial burden on their professional and personal lives, making dialysis patients more likely to experience significant impairments in health‐related quality of life [38, 65]. It has been also reported that physical limitations in daily activities tend to increase with aging [66]. Therefore, It is possible that there is a strong tendency for the patients undergoing dialysis to believe that achieving a HLE is difficult, regardless of individual or family efforts. Additionally, the fact that the dialysis group in this study had a higher proportion of individuals living alone and males may also be related to these results. The high proportion of individuals living alone suggests that they may have less support in daily life, leading many respondents to perceive that, regardless of their own efforts or those of their (non‐cohabiting) family members, difficulties in daily life would persist. Furthermore, it has been suggested that among dialysis patients, men, compared to women, tend to have stronger correlations between cognitive appraisal, to rely more on cognitive styles like self‐control and personality traits such as high self‐esteem when facing adversity, and to use more instrumental and control‐related coping strategies; consequently, the effects of dialysis, including loss of autonomy and control, may significantly impact their coping ability, leading to higher stress levels and poorer QoL [67]. This may have been a factor in why dialysis patients in this study perceived achieving HLE as difficult. Furthermore, we considered that the low SOC score might have influenced the negative perception of achieving HLE. However, although the overall average SOC score of the participants in this study was 53.50, which was lower than the values reported in previous studies, there was no significant difference among the three groups. Therefore, it is difficult to fully explain the dialysis patients' negative perception of achieving HLE based solely on this factor. This study was unable to clarify the detailed factors influencing the perception of achieving HLE. However, the results suggest that perceptions of HLE may vary depending on the specific disease. To increase the number of individuals who achieve HLE regardless of the presence or absence of disease, it is necessary to conduct further investigations with a greater variety of diseases and disabilities to have better understanding of perception of HLE and to suggest specific support measures.

Among individuals with diseases (cancer and dialysis groups), being female, having cohabitants, and having a higher SWLS score are significantly associated with achieving a HLE. Interestingly, within the disease groups, HLE‐subgroup (those who responded “no” to the question “Are you currently affected in your daily life because of health problems?”) has higher SWLS socores than the average score of Japanese general population (average SWLS score of 21.99). Although numerous studies suggest that life satisfaction has negative association with lower physical, cognitive, and psychological health [53, 54, 55, 57, 58, 68, 69], extensive body of research also has indicated its relationship with how people perceive their health (i.e., subjective evaluation of their health) and themselves. For example, good self‐perception on ageing life [57] and high subjective health status including physical and cognitive abilities [70]; Lopez‐Ortega et al., 2016; [54] have positive association with life satisfaction whereas low subjective health status and well‐being [71], low QOL [52], the perception of having poor health than their peers, and the perception of being ignored/hated due to old age [58] can have negative association with life satisfaction. An international study with patients with Parkinson's disease reports that SWLS could discriminate subjectively perceived healthy and unhealthy patients, subjectively perceived disabled and nondisabled patients, and those with subjectively perceived mild versus sever impact of disability on their lives [53]. These previsou studies suggest that even among persons who live with a disease, there are certain amount of people who feel that they are healthy and their health status does not affect much in their daily lives (i.e., people who achive a HLE). We believe that increasing the number of such individuals is one way to extend HLE and the present study indicates that high level of life satisfaction may be one of the contributing factors.

To increase life satisfaction, the important role of social participation and social supports have been pointed out. Prior studies in a Japanese population suggest that social participation in a community, hobby, volunteer activities, sports group or organization can have positive impact on decreasing the risk of disability and mental health in middle‐aged adults and elders [72, 73, 74, 75], and offering meaningful roles within organizations may also have positive impact on mental health especially for men [74]. Individual relationship with friends and family can be a protective role against psychological distress in older women living alone [72]. In addition, the positive relationship between the social supports and one's life satisfaction has been shown in the patients with diseases and disabilities including spinal cord injury [54], breast cancer [76], and chronic obstructive pulmonary disease [77].

Contrary to expectations, “having someone close to talk to” was associated with a decreased likelihood of reporting no impact on daily life due to health problems. However, as mentioned earlier, many previous studies have reported that social support contributes to increased life satisfaction, and the results of this study partially contradict that. One possible reason is that even when social support exists, its quality or the effectiveness for the recipient may not be sufficient. Tsuji & Khan [78] pointed out that among elderly people in Japan, the perception of social support differs depending on factors such as gender, life history, and social roles. Moreover, the balance and reciprocity between the provider and the recipient of support greatly influence its meaning. These findings suggest that a single question such as “do you have someone close to talk to?” may not adequately capture the diverse nature and actual state of social support. In other words, having someone close to talk to may not necessarily be perceived as support that contributes to achieving a healthy life expectancy (HLE). It is also possible that there are cases where individuals experience difficulties or obstacles in daily life and have someone to talk to about it, but this has not led to the realization of HLE. Therefore, it is necessary to evaluate the actual state of social support, including elements such as the content and quality of support, timing of its provision, and the recipient's perception. It will be important to examine the relationship between social support and healthy life expectancy from a more multifaceted perspective, with attention to the qualitative aspects of how people living with illness or disability receive and interpret the support they get.

In addition, the effects of psychological and psychoeducational interventions have also been noted to improve life satisfaction and QOL for those living with diseases [79]. In particular, dialysis patients may benefit interventions that target affect factors, particularly depression, stress, and cognititions such as patients’ belief in self‐control and problem solving [67]. Cancer patients, who generally experience psychological symptoms such as anxiety, depression, and fear of reccurance, may benefit from psychological interventions, including cognitive behavioural therapy, mindfulness, and relaxation [80]. A systematic psychotherapeutic intervention has been shown to be effective to alleviate depressive symtoms in patinets with advanced cancer [81].

There are several limitations in this study. Firstly, our online cross‐sectional survey does not allow for the establishment of a causal relationship between disease affliction and health concepts. Secondly, due to the complexity of the analysis and limited number of subjects, this study did not consider the severity of the diseases in the cancer and dialysis groups. Lastly, the study's findings are specific to colorectal cancer and kidney failure undergoing dialysis and can not be generalized to other diseases or disabilities. To have better understanding of the perception of HLE of individuals with diseases and disabilities, further studies which include greater variety of diseases and disabilities are necessary. Neverthless, we belive that this study has social significance in that it explored the perception of HLE among Japanese individuals with diseases and disabilities—an area that has been largely unexplored—and suggested a possible relationship with life satisfaction. This may provide insights for measures aimed at reducing the gap between life expectancy and HLE.

## Author Contributions

**Kyunghee Lee:** conceptualization, data curation, formal analysis, visualization, writing – original draft, methodology, investigation, writing – review and editing, validation, software, resources; **Kazumi Ota:** conceptualization, data curation, formal analysis, visualization, writing – original draft, methodology, investigation, writing – review and editing, validation, resources; **Tetsuya Toma:** conceptualization, supervision, project administration, funding acquisition, validation, resources; **Masako Toriya:** conceptualization, supervision, project administration, validation, funding acquisition, resources.

## Conflicts of Interest

The authors declare no conflicts of interest.

## Supporting information


**Figure 1:** Histogram of Q6‐6 and Q6‐7 scores in the three groups (cancer, dialysis, and ND groups). **Figure 2:** Histograms of SWLS scores for Non‐HLE and HLE sub‐groups in Cancer, Dialysis, ND groups.

**Table1:** Questionnaire. Supplementary Table2 Allocation of the subjects.

## Data Availability

The data supporting the findings of this study were obtained from a third‐party data provider (Macromill Inc.) under contractual agreements that preclude public data sharing due to privacy and proprietary restrictions. Deidentified datasets may be available to qualified researchers upon reasonable request to the corresponding author, subject to approval from the data provider and adherence to applicable data use agreements.
