# Supplementary material for: Perceptions of “Healthy Life Expectancy” of Individuals With Diseases: An Online Survey in Japan
Source: Health Sci Rep. 2025 Nov 26;8(12):e71533. doi: 10.1002/hsr2.71533 (PMC12657619; doi:10.1002/hsr2.71533)
Supplement: Supplementary file 1 — Figure 1: Histogram of Q6‐6 and Q6‐7 scores in the three groups (cancer, dialysis, and ND groups). Figure 2: Histograms of SWLS scores for Non‐HLE and HLE sub‐groups in Cancer, Dialysis, ND groups. [file HSR2-8-e71533-s001.pdf]

Supplementary Figure1 Histogram of Q6-6 and Q6-7 scores in the three groups (cancer, dialysis, and ND groups)

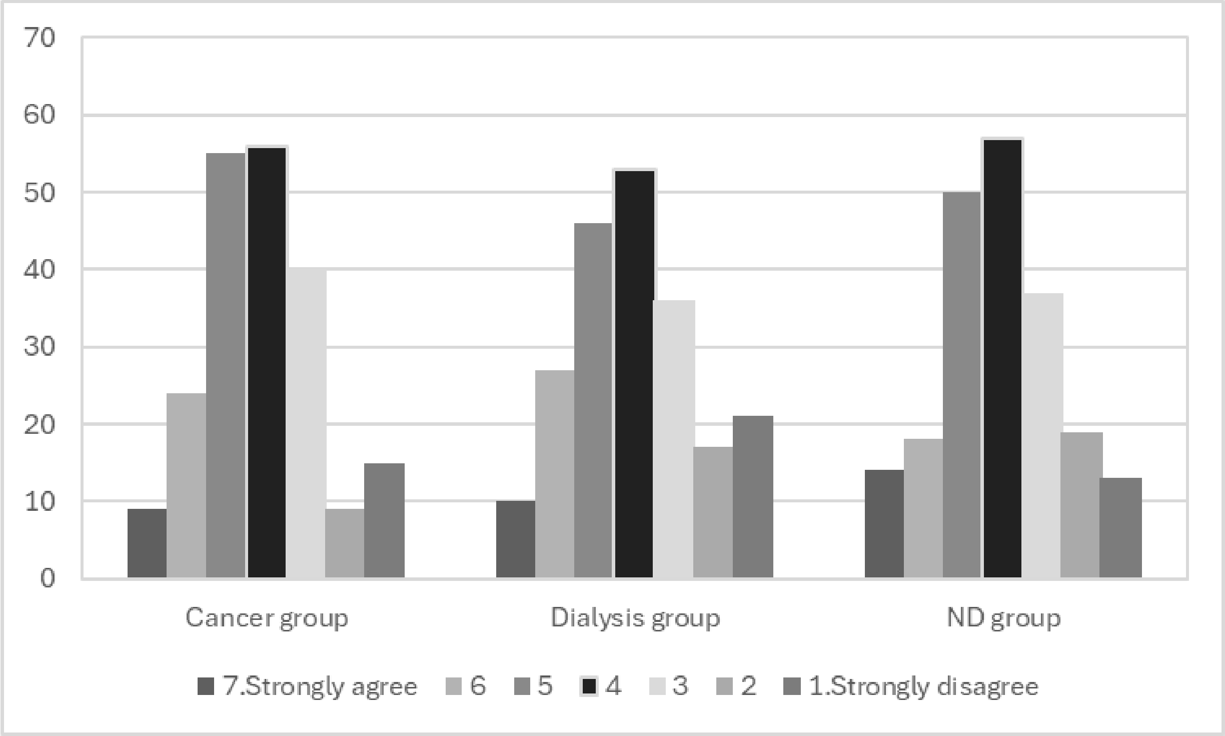

Q6-6: I think this definition can be achieved even if one has a physical disease or disability.

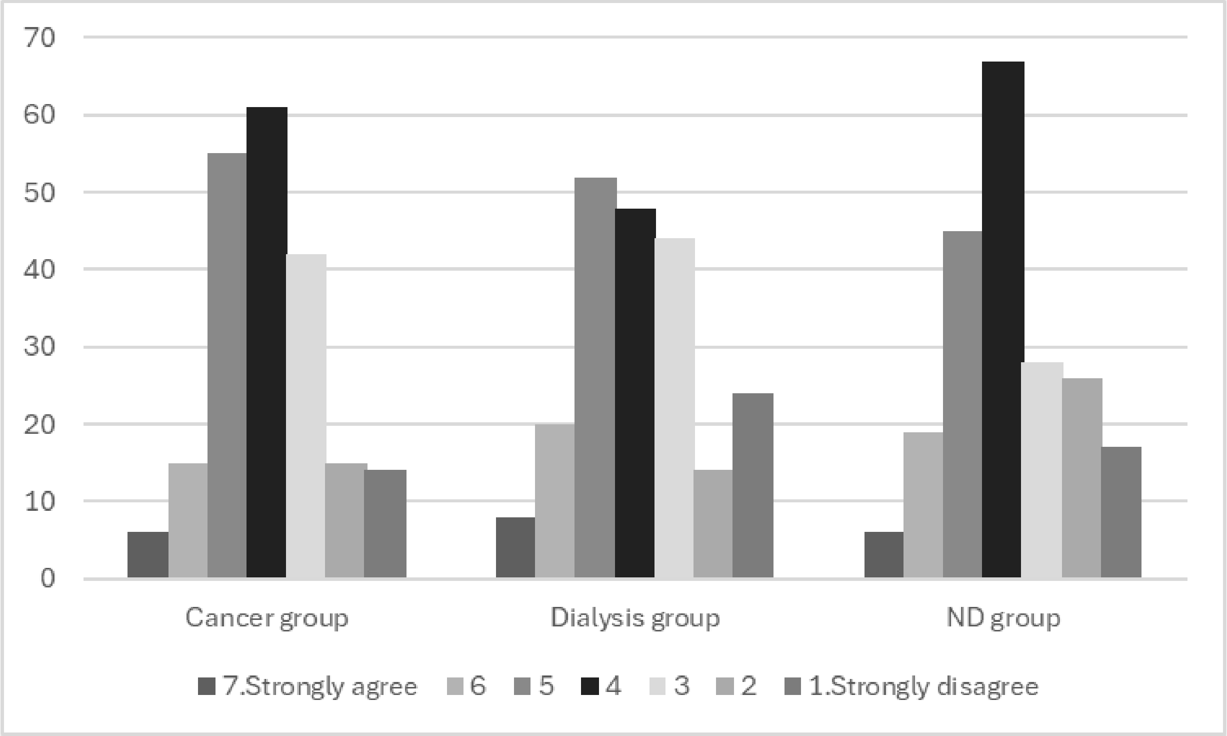

Q6-7: I think this definition can be achieved even if one has a mental disease or disability.

Supplementary Figure2 Histograms of SWLS scores for Non-HLE and HLE sub-groups in Cancer, Dialysis, ND groups

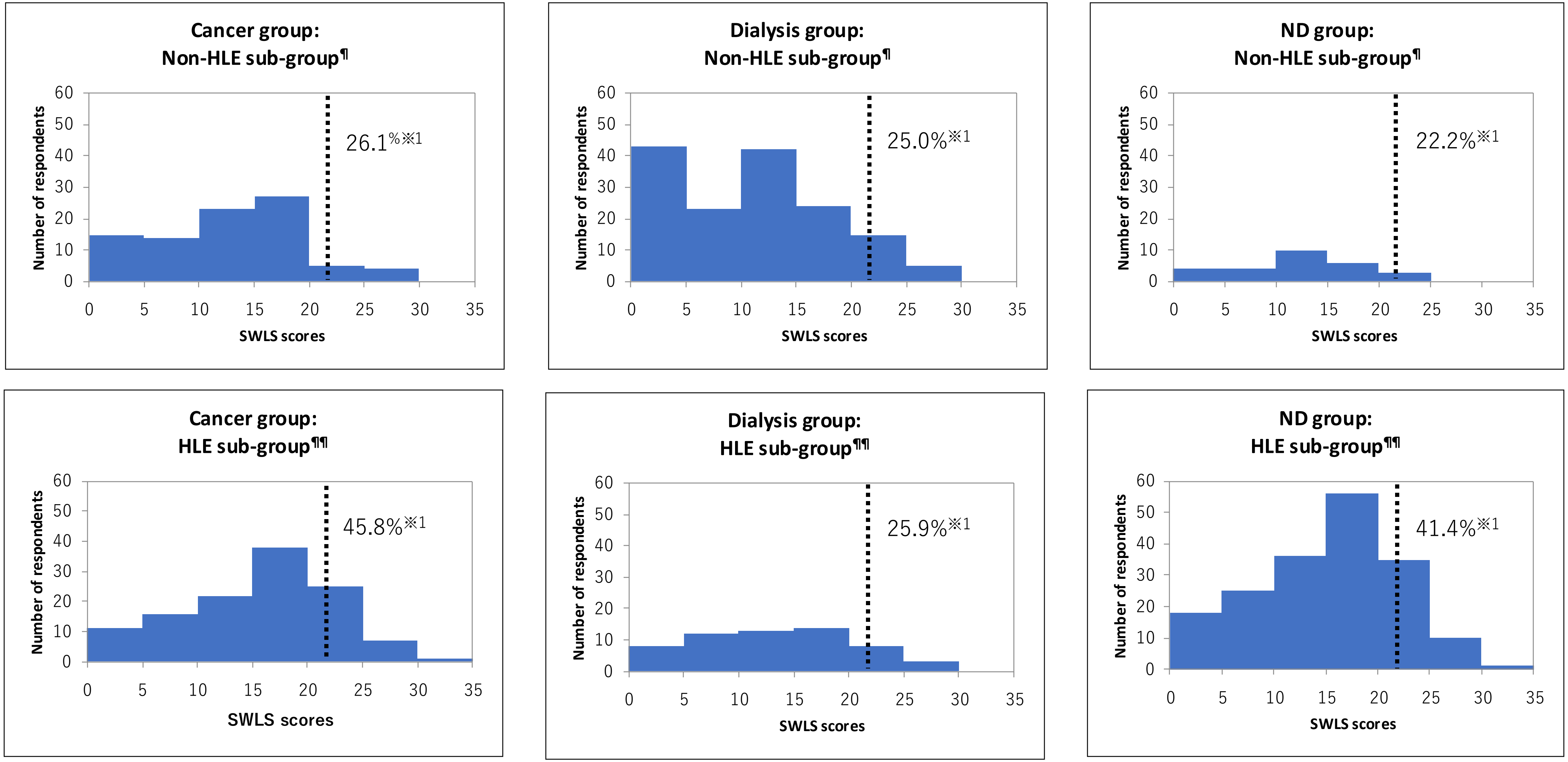

¶: Non-healthy life expectancy sub-group

¶¶: Healthy life expectancy sub-group

······ : the average SWLS score (21.99) reported in Sumino, 1991

※1: Percentage of respondents who scored over 21.99 in each sub-group.
