# Supplementary material for: Perceptions of “Healthy Life Expectancy” of Individuals With Diseases: An Online Survey in Japan
Source: Health Sci Rep. 2025 Nov 26;8(12):e71533. doi: 10.1002/hsr2.71533 (PMC12657619; doi:10.1002/hsr2.71533)
Supplement: Supplementary file 2 — Table1: Questionnaire. Supplementary Table2 Allocation of the subjects. [file HSR2-8-e71533-s002.pdf]

Supplementary Table1 Questionnaire

	Category	Subcategory	Instructions	Questions	Answer options	Note
Screening survey						
SC1	Screening items	SC1-1	Read the sentences below and select all that apply. (Multiple selections possible)	—	1=Have received treatment for colorectal cancer (surgery, drug treatment (chemotherapy), radiation therapy, etc.) within the past 3 years /2=Currently undergoing dialysis treatment /3=Have been diagnosed with dementia or a mental disease / 4=Do not currently have any disease / 5=None of the above	1=Cancer group 2=Dialysis group 4=ND group Those who chose 3, 5 or several answers were not included in the study
Primary survey						
Q0	Demographic (only the cancer patients)	Q0-1	Read the sentence below and select all that apply.	What stage of colorectal cancer was it when you received colorectal cancer treatment within the last 3 years? (Choose only one)	Stage 0 (the cancer remains within the colon mucosa) / Stage 1 (the cancer has grown through the mucosa of the colon and invaded the muscle layer) / Stage 2 (The cancer has grown beyond the mucosa of the colon but has not spread to the lymph nodes) / Stage 3 (The cancer has spread to the lymph nodes near the colon, it has not spread further) / Stage 4 (The cancer has spread outside of the colon and has	—

					been carried through the lymph and blood systems to distant parts of the body) / unknown	
Q1	Demographics	Q1-1	Read the sentences below and select all that apply.	What is your current living condition? (Choose only one)	I have a family living with me/ I have a family but live alone/I don't have a family and live alone/ I don't have a family and live with someone/ Other	—
		Q1-2	—	Which educational institution did you last graduate from (including those that dropped out) or are you currently attending? (only one)	Junior high school / high school / vocational school / technical college · junior college / university / graduate school / other (FA)	—
		Q1-3	—	Do you have someone close to whom that you can talk if you are suffering from a disease or	Yes / No	—

				disability?		
		Q1-4	—	Are you currently taking care of someone (childcare or nursing care)? (Multiple selections possible)	Child / Parent or grandparent / Spouse / Other / Nobody (not taking care of anyone)	—
Q2	Subjective health	Q2-1	Read the sentence below and select all that apply.	Are you currently affected in your daily life due to health problems?	Yes / No	—
Q3	Sense of Coherence 13-item instrument (SOC-13)	Q3-1	Read the sentences below and select all that apply.	Do you have the feeling that you don’t really care about what goes on around you?？	1=Very seldom or never to 7=Very often	—
		Q3-2	—	Has it happened in the past that you were surprised by the behaviour of people whom you thought you knew well?	1=Never happened to 7=Always happened	—
		Q3-3	—	Has it happened that people whom you counted on disappointed you?	1=Never happened to 7=Always happened	—

	Q3-4	—	Until now your life has had:	1=No clear goals or purpose at all to 7=Very clear goals and purpose	—
	Q3-5	—	Do you have the feeling that you’re being treated unfairly?	1=Very often to 7=Very seldom or never	—
	Q3-6	—	Do you have the feeling that you are in an unfamiliar situation and don’t know what to do?？	1=Very often to 7=Very seldom or never	—
	Q3-7	—	Doing the things, you do every day is:	1=A source of deep pleasure and satisfaction to 7=A source of pain and boredom	—
	Q3-8	—	Do you have very mixed-up feelings and ideas?	1=Very often to 7=Very seldom or never	—
	Q3-9	—	Does it happen that you have feelings inside you would rather not feel?	1=Very often to 7=Very seldom or nev	—
	Q3-10	—	Many people – even those with a strong character – sometimes feel like sad sacks (losers) in certain situations. How often have you felt this way in the past?	1=Never to 7=Very often	—

		Q3-11	—	When something happened, have you generally found that:	1=You overestimated or underestimated its importance to 7=You saw things in the right proportion	—
		Q3-12	—	How often do you have the feeling that there’s little meaning in the things you do in your daily life?	1=Very often to 7=Very seldom or never	—
		Q3-13	—	How often do you have feelings that you’re not sure you can keep under control?	1=Very often to 7=Very seldom or never	—
Q4	Satisfaction with Life Scale (SWLS)	Q4-1	Read the sentences below and select all that apply.	In most ways my life is close to my ideal.	1=Strongly agree to 7=Strongly disagree	—
		Q4-2	—	The condition of my life is excellent.	1=Strongly agree to 7=Strongly disagree	—
		Q4-3	—	I am satisfied with my life.	1=Strongly agree to 7=Strongly disagree	—
		Q4-4	—	So far I have gotten the important things I want in life.	1=Strongly agree to 7=Strongly disagree	—
		Q4-5	—	If I could live my life over, I would change almost nothing.	1=Strongly agree to 7=Strongly disagree	—
Q5	Awareness of healthy life expectancy	Q5-1	Read the sentence below and select all that apply.	Regarding the term "healthy life expectancy", please select	1=I know the meaning well / 2=I kind of know the meaning / 3=I've heard of it, but I	—

				the one that applies to you.	don't know the meaning / 4=I've never heard of it	
Q6	Perceptions on healthy life expectancy	Q6-1	MHLW defines healthy life expectancy as "the average number of years a person is expected to live without any limitations on his or her daily activities", and promote its extension.	I think this definition can be achieved through individual effort.	1=Strongly agree to 7=Strongly disagree	—
		Q6-2	—	I think this definition can be achieved through the efforts of the family surrounding the individual.	1=Strongly agree to 7=Strongly disagree	—
		Q6-3	—	I think this definition can be achieved through the efforts of friends surrounding the individual.	1=Strongly agree to 7=Strongly disagree	—

Q6-4	—	I think this definition can be achieved through the efforts of companies and educational institutions surrounding the individual.	1=Strongly agree to 7=Strongly disagree	—
Q6-5	—	I think this definition can be achieved through the efforts of the national and local governments surrounding individuals.	1=Strongly agree to 7=Strongly disagree	—
Q6-6	—	I think this definition can be achieved even if one has a physical disease or disability.	1=Strongly agree to 7=Strongly disagree	—
Q6-7	—	I think this definition can be achieved even if one has a mental disease or disability.	1=Strongly agree to 7=Strongly disagree	—

Supplementary Table2 Allocation of the subjects

	Cancer group			Dialsys group			ND group		
	Total	Male	Female	Total	Male	Female	Total	Male	Female
Total	208	107(51.4)	101(48.6)	210	160(76.2)	50(23.8)	208	104(50.0)	104(50.0)
40s n(%)	43	23(53.5)	20(46.5)	41	27(65.9)	14(34.1)	52	26(50.0)	26(50.0)
50s n(%)	57	29(50.9)	28(49.1)	70	56(80.0)	14(20.0)	52	26(50.0)	26(50.0)
60s n(%)	56	29(51.8)	27(48.2)	58	45(77.6)	13(22.4)	52	26(50.0)	26(50.0)
70s+ n(%)	52	26(50.0)	26(50.0)	41	32(78.0)	9(22.0)	52	26(50.0)	26(50.0)
